# Knockdown of lncRNA-PANDAR suppresses the proliferation, cell cycle and promotes apoptosis in thyroid cancer cells

**DOI:** 10.17179/excli2017-113

**Published:** 2017-03-23

**Authors:** Zhirong Li, Bo Gao, Shuai Hao, Wuguo Tian, Yi Chen, Lingli Wang, Xiaohua Zhang, Donglin Luo

**Affiliations:** 1 Department of Breast and Thyroid Surgery, Research Institute of Surgery, Daping Hospital, Third Military Medical University, Chongqing 400042, China

**Keywords:** thyroid cancer, lncRNAs, PANDAR, proliferation, cell cycle, apoptosis

## Abstract

Long non-coding RNAs (lncRNAs) have been found to show important regulatory roles in various human cancers. Lnc-RNA PANDAR is a novel identified lncRNA that was previously reported to show abnormal expression pattern in various cancers. However, little is known of its expression and biological function in thyroid cancer. Here, we used the quantitative real-time PCR (qRT-PCR) to determine the expression of PANDAR in 64 thyroid cancer tissues. We found that expression of PANDAR was up-regulated in thyroid cancer tissues compared with adjacent non-tumor tissues. Functional assays *in vitro* demonstrated that knockdown of PANDAR could inhibit proliferation, cell cycle progression, induces the apoptosis, inhibit invasion of thyroid cancer cells. Thus, our study provides evidence that PANDAR may function as a potential target for treatment for patients with thyroid cancer.

## Introduction

Thyroid cancer (TC) is originated from follicular or parafollicular thyroid cells which is one of the most common malignant tumors of endocrine organs and has been steadily increasing in morbidity and mortality over the years (Vuong et al., 2017[20]). The incidence of TC has been increased by an average of 4.5 % per year from 2007 to 2011 in United States, and has ranked the eighth most frequent cancer in China. Thus the rapid increase in TC incidence poses a substantial burden (Chen et al., 2016[2]; Siegel et al., 2016[19]; Wang and Wang, 2012[23]). Previous studies refer that radiation, chemotherapy, and surgery are the common treatment methods for thyroid cancer, but they all produce poor satisfaction (Hay et al., 2013[8]; Nagaiah et al., 2011[14]). In addition to environmental and genetic predisposing factors, increasing studies have shown that epigenetic alteration may play pivotal roles in the development and progression of various kinds of tumors including thyroid cancer (Feinberg et al., 2006[4]; Zhang et al., 2008[25]). 

LncRNAs, which act as an important component of tumor biology, are involved in a number of regulatory functions including modulation of apoptosis and invasion, reprogramming of induced pluripotent stem cells, marker of cell fate and parental imprinting (Halley et al., 2014[6]; Li and Wang, 2012[11]). And it is well recognized that some altered expression of lncRNAs has been frequently linked with cancer pathogenesis (Hu et al., 2016[9]; Li et al., 2014[10]; Prensner et al., 2011[16]; Wang et al., 2015[21][22]), providing new insight into the genetic and molecular mechanisms of cancer. PANDAR, promoter of CDKN 1A antisense DNA damage activated RNA, is a novel non-coding RNA mapping to 6p21.2, which underlies metastatic progression and chromosomal instability in multiple cancer. Recently, Han et al. (2015[7]) reported that lncRNA PANDAR was induced in a p53-dependent manner and interacts with the transcription factor NF-YA to limit the expression of pro-apoptotic genes. Moreover, lncRNA PANDAR regulates the G1/S transition of breast cancer cells by suppressing p16 (INK4A) expression (Sang et al., 2016[17]). And PANDAR functions as an oncogene in bladder cancer, promoting cellular proliferation, migration and apoptosis (Zhan et al., 2016[24]). Furthermore, Han et al. demonstrated that low expression of long non-coding RNA PANDAR predicts a poor prognosis of non-small cell lung cancer and affects cell apoptosis by regulating Bcl-2 (Han et al., 2015[7]). However, the expression and detailed function of PANDAR in thyroid cancer remains largely unknown and needs to be investigated.

In this study, we explored the expression pattern of PANDAR in thyroid cancer tissues and thyroid cancer cell lines. Furthermore, the biological function of lncRNA-PANDAR in thyroid cancer cell^,^ proliferation, cell cycle and apoptosis was examined *in vitro.*

## Materials and Methods

### Patients and tissue samples of TC

Sixty-four paired samples of TC and adjacent non-tumor tissues (more than 2 cm away from the tumor) were obtained from patients who received surgery in Daping Hospital, Third Military Medical University. Tissue samples were collected, immediately snap frozen in liquid nitrogen and stored at −80 °C until further analysis. Before the use of these clinical materials for research purposes, informed consents were provided by all patients and approval of the Ethics Committee of Daping Hospital, Third Military Medical University was obtained. The experiment protocol was approved by the Ethics Committee of clinical laboratory.

### Cell culture and siRNA transfection

Normal human thyroid follicular epithelial cell Nthy-ori3-1 cell and human thyroid cancer K-1, TPC-1, SW579, FTC133 and XTC-1 purchased from American Type Culture Collection (ATCC) were cultured in RPMI-1640 medium with 10 % fetal bovine serum (FBS), 0.1 mM non-essential amino acids, 1 mM sodium pyruvate, and 1 % penicillin-streptomycin (Sigma, USA) in the humidified incubator with 5 % CO_2_ at 37 °C. Cells were grown in monolayer and conventionally passaged when cell attachment rate reached 90 %.

The PANDAR-siRNA expression vector was constructed based on the full length of wild-type lncRNA-PANDAR coding sequence by Genechem Biotech (Shanghai, China). The target sequence for siRNA- PANDAR vector construction was antisense: PANDAR-1: 5'-GCAATCTACAACCTGTC TT3', PANDAR-1:5'TTTCGAACGGAAC AGAGACUUAUACAGATT-3'. SiRNA vector with no silenced PANDAR sequence (si-NC) was transfected into thyroid cancer cells as the controls. Cells were plated and cultured in growth media until cell density reached 70 % prior to siRNA transfection and cell transfections were conducted with Lipofectamine 2000 reagent based on the manufacturer's protocol (Invitrogen, USA). Cells were harvested after 48 h for qRT-PCR and Western blot analyses.

### Cell viability assay

Cell viability was detected using 3-(4,5-dimethyl-2-thiazolyl)-2,5-diphenyltetrazolium bromide (MTT) assay as previously described (Cohen et al., 2002[3]). Briefly, after being transfected with siRNA-PANDAR or control siRNA vector for 24 h, thyroid cancer cells were trypsinized and plated in 96-well plates at a density of 5×10^3^ cells/well. After 24 h of cultivation, supernatant was abandoned, and 20 μL of MTT was allowed to be added in for another 4-h incubation in RPMI-1640 supplemented with 10 % FBS. Consequently, 150 μL of dimethylsulfoxide (DMSO) was mixed with the cells for 10 min. Absorbance of cells in each well was observed at 570 nm under an absorption spectrophotometer (Olympus, Japan) for the cell number calculation.

### Clonogenic assay

Clonogenic assay was performed with a modification of previously published method (Franken et al., 2006[5]). In brief, after completion of siRNA transfection, cells were plated into the 6 wells plate in triplicate and at a cell density of 100 cells/well. Then, the cells were grown in RPMI-1640 containing 10 % FBS for 14 days. After that, the cells were fixed and stained with Crystal violet, followed with air-dry. Finally, colonies were counted under microscope (IX83, Olympus), and cell number each colony was at least 30 cells.

### Cell apoptosis and cell cycle assay

After transfection with siRNA, cells were cultured in RPMI-1640 containing 10 % FBS for another 48 h, and then, the cells were trypsinized and apoptosis was detected using the Annexin V-FITC Apoptosis Detection Kit (BD, USA). Consequently, cells were pelleted and washed with cold PBS suspended with cold PBS. The TPC and SW579 cells were then treated with Annexin V-propidium iodide (PI) in the dark at room temperature according to the manufacturer's instructions. The cells were kept on ice in the dark and immediately analyzed by flow cytometry (FACSCalibur, BD Biosciences) and the data were analyzed using the Cell Quest software. For cell cycle analysis, TPC and SW579 cells transfected with Si-NC or Si-PANDAR were harvested for 48 h. Then cells were fixed with 70 % ethanol at −20 °C for 12 h and stained with propidium iodide (PI) (Sigma) in the presence of Ribonuclease A (Takara Biotechnology, Dalian, China) for 30 min at room temperature. The cell cycle distribution was analyzed by flow cytometry (FACSCalibur, BD Biosciences). All the experiments were repeated three times.

### Quantitative Real-time PCR

Total RNA was isolated from thyroid cancer tissues and thyroid cancer cell lines using TRIzol Reagent (Invitrogen). RNA was reverse transcribed using SuperScript First Strand cDNA System (Invitrogen) according to the manufacturer's instructions. Quantitative PCR was conducted using SYBR Green Taq Mix (Takara) on a Bio-Rad Real-Time PCR System, and GAPDH was used as internal control for normalization. The primers were synthesized by Invitrogen (Shanghai, China). Their sequences were as follows: PANDAR primers, forward: 5′-CTGTTAAGGTGGTGGCATTG-3′, reverse: 5′-GGAGGCTCATACTGGCTGAT-3′; GAPDH primers, forward: 5′- CGCTCT CTGCTCCTCCTGTTC-3′, reverse: 5′-ATCCGTTGACTCCG ACCTTCAC-3′. Experiments were repeated at least three times.

### Western blotting 

Protein homogenates were prepared from thyroid cancer cell lines. Equal amounts of protein (50 μg) were separated by SDS/PAGE (10 % gel), transferred on to nitrocellulose membranes and blocked by 5 % non-fat milk. The membranes were incubated with primary antibodies at 4 °C for 12 h, and then were incubated with horse radish peroxidase (HRP)-conjugated secondary antibodies at room temperature for 1 hour. The antibody-antigen reactions were visualized by using the ECL Plus Western Blotting Detection System (GE Healthcare, Piscataway, NJ). The density of blots was analyzed by ImageJ software (National Institutes of Health, Bethesda, MD). The antibodies were purchased from Cell Signaling Technology and were used at manufacturer-recommended dilutions. 

### Statistical analysis

All data in this study are expressed as mean ± SD, and differences between groups were determined using analysis of variance (ANOVA) with SPSS version 18.0. P < 0.05 was considered statistically significant. 

## Results

### LncRNA-PANDAR expression was upregulated in thyroid tissues and cell lines

**Figure 1:**
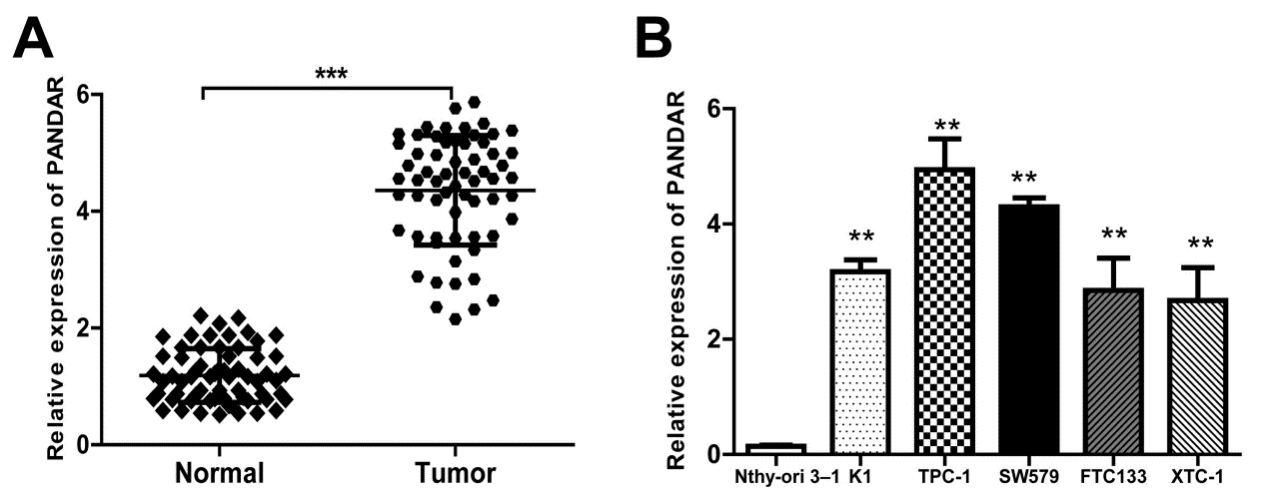
LncRNA PANDAR expression is increased in thyroid cancer tissues and cell lines. A. Relative expression of PANDAR in 64 pairs of thyroid cancer tissues and adjacent non-tumor tissues by qRT-PCR analysis. ***P<0.001 compared with non-tumor control. B. The expression levels of PANDAR in a panel of thyroid cancer cell lines were determined by qRT-PCR and compared with that in human normal thyroid cells (Nthy-ori 3-1). **P < 0.01 compared with the Nthy-ori 3-1 cell. Data represent the mean ± SD from three independent experiments. *P < 0.05; **P < 0.01.

To investigate the roles of PANDAR in thyroid pathogenesis, expressions of PANDAR in thyroid cancer tissue (a total of 64 patients) and in five kinds of cell lines were detected using qRT-PCR (Figure 1(Fig. 1)). Results showed that the relative PANDAR mRNA expression in thyroid cancer tissues was significantly high compared to that in adjacent normal tissues (Figure 1A(Fig. 1), P < 0.001). Interestingly, relative PANDAR expression in five kinds of cell lines (K1, TPC-1, SW579, FTC133, XTC-1) were all significantly higher than that in normal cell line Nthy-ori 3-1 (Figure 1B(Fig. 1), P < 0.01).

### Knockdown of PANDAR impairs proliferation and invasion of TPC-1 and SW579 cells in vitro

As shown in Figure 1B(Fig. 1), PANDAR expression in TPC-1 and SW579 was comparatively higher than that of the other three kinds of cell lines. Therefore, we selected TPC-1 and SW579 cell lines as a model to investigate the effect of PANDAR on cell proliferation and apoptosis. 

**Figure 2:**
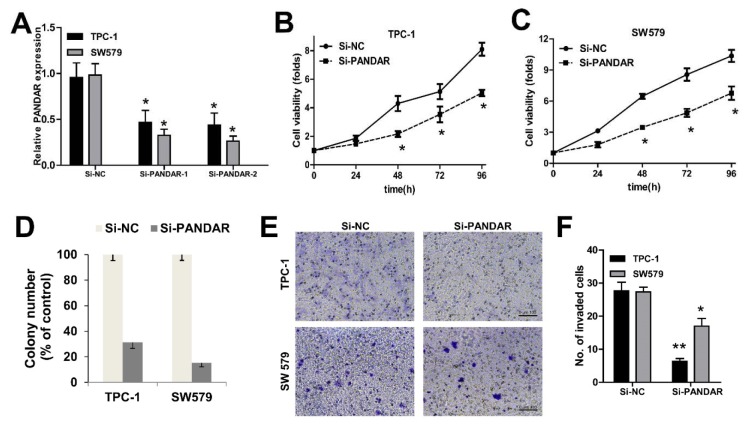
The effect of PANDAR expression on cell viability and proliferation of thyroid cancer cells. A. qPCR analysis of PANDAR expression levels following the treatment of TPC-1 and SW579 cells with siRNAs against PANDAR. B and C. MTT assay showed knockdown of PANDAR inhibited two types of thyroid cancer cells' viability. D. Soft-agar assay measuring colony formation of PANDAR knockdown cells. E. Cell invasion of the two types of thyroid cancer cells measured with transwell assay (scale bar: 100 μm). F. Cell count of the invaded cells (per view) based on the transwell invasion assay. Colony number was normalized to that obtained with cells transfected with Si-NC, which was set to 100%. Silencing of PANDAR significantly decreased the colony-forming ability. Each assay was performed in triplicate. Data are mean ± SD. * P < 0.05, **P < 0.01.

We knocked down PANDAR expression in TPC-1 and SW579 cancer cells by transfection with NC-siRNAs, si-PANDAR. As shown in Figure 2A(Fig. 2), cells transfected with si-PANDAR presented a significantly decreased mRNA expression level of PANDAR compared with the Si-NC group in both cells by qRT-PCR (P < 0.05; Figure 2A(Fig. 2)). To determine the effect of PANDAR on the viability and proliferation of thyroid cancer cell *in vitro,* MTT assays showed that knockdown of PANDAR obviously suppressed the viability of TPC-1 and SW579 in a time dependent manner (P < 0.05; Figure 2B-D(Fig. 2)). The ability to form colonies by TPC-1 and SW579 cells was also suppressed significantly after knockdown of PANDAR when compared with that by the negative controls (P < 0.01; Figure 2D(Fig. 2)). These results showed that PANDAR depletion had an obvious inhibitory effect on the growth of thyroid cancer cells. Furthermore, knockdown of PANDAR resulted in attenuated invasion of TC cells measured by transwell invasion assay (Figure 2E, F(Fig. 2)).

### Knockdown of PANDAR suppressed cell cycle progression of thyroid cancer cells

**Figure 3:**
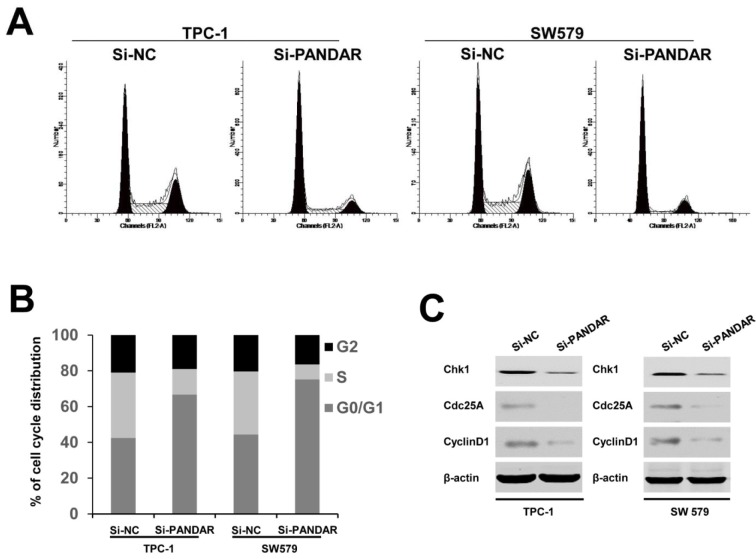
Effect of PANDAR knockdown on cell cycle. A. Cell cycle distribution was measured by propidium iodide staining followed by flow cytometry. B. The two cells had cell-cycle arrest at the G0-G1 phase compared with cells transfected with Si-NC. C. Western blotting was used to detect the protein expression of Chk1, Cdc25A and CyclinD1; β-actin was used as control. Each assay was performed in triplicate. Data are mean ± SD. *P < 0.05, **P < 0.01.

Having found the effect of PANDAR downregulation on the proliferation of thyroid cancer cell lines, we then examined the impact of decreased expression of PANDAR on cell cycle in thyroid cancer cells. Flow cytometric analysis showed a decrease in the percentage of cells in the S phase and a marked accumulation in the percentage of cells in the G0/G1 phase in the Si-PANDAR groups in TPC-1 and SW579 cells (Figure 3A and B(Fig. 3)), compared with the Si-NC groups. Accordingly, to verify whether PANDAR could indeed arrest the cell cycle in G0/G1 stage or not, cell cycle-related protein expression including cyclin D1, Chk1, and Cdc25A were analyzed. These cell cycle regulatory proteins were significantly decreased when cells were transfected with Si-PANDAR compared to that in controls (Figure 3C(Fig. 3)). Taken together, these results illuminated that PANDAR may function as an oncogene involved in the stimulation of thyroid cancer cell proliferation.

### Knockdown of PANDAR increased the apoptosis of thyroid cancer cells

**Figure 4:**
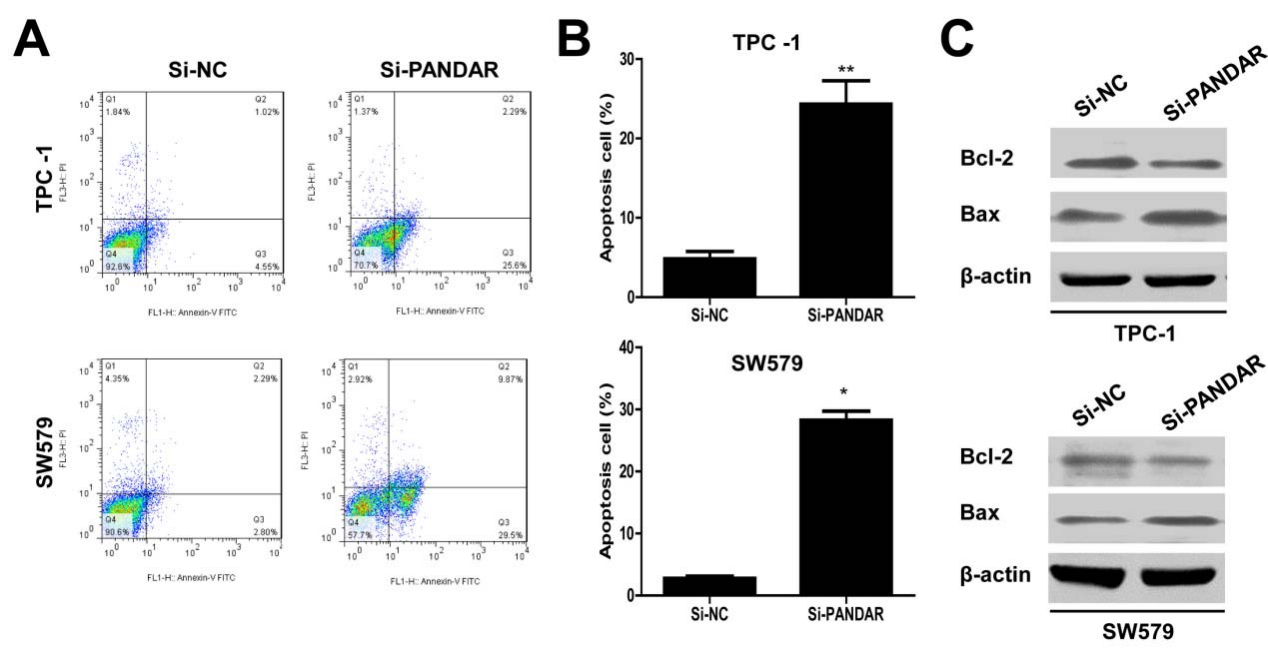
Downregulated lncRNA PANDAR increased the apoptosis of thyroid cancer cells. A. Apoptosis of TPC-1 and SW579 cell lines was determined by flow cytometry. B. Histogram of percentage of apoptotic cells, according to (A). C. Western blotting was used to detect the protein expression of Bcl-2 and Bax; β-actin was used as control. Each assay was performed in triplicate. Data are mean ± SD. *P < 0.05, **P < 0.01

As shown by flow cytometry analysis in Figure 4A and B(Fig. 4), compared with the cells transfected with si-NC, si-PANDAR treatment caused increased apoptosis in TPC-1 and SW579 cells significantly (P < 0.01, TPC-1 and P < 0.05, SW579). Moreover, we examined Bcl-2 and Bax expression in thyroid cancer cells in response to PANDAR knockdown. As is well known, Bcl-2 is recognized to inhibit apoptosis and facilitate resistance to multifarious apoptosis-inducing factors. While, Bax could inhibit the anti-apoptotic effect of Bcl-2 by forming a heterodimer with Bcl-2. Western blotting analysis indicated that PANDAR knockdown suppressed the protein expression of Bcl-2 and in contrast enhanced the protein expression of Bax in TPC-1 and SW579 cells (Figure 4C(Fig. 4)).

## Discussion

Recently, increasing evidence has suggested that lncRNAs may play vital regulatory roles in the development of various types of cancers (Cheetham et al., 2013[1]; Shore et al., 2012[18]). A number of papers have shown that PANDAR show abnormal expression pattern in various cancers (Lu et al., 2017[12]; Peng and Fan, 2015[15]; Zhan et al., 2016[24]), however, few have reported the potential roles of lncRNA PANDAR in thyroid cancer. In the present study, we found that PANDAR was highly expressed in thyroid cancer tissues and cell lines. In addition, loss of function assay revealed that knockdown of PANDAR significantly impaired the proliferation and invasion, and also arrested the cell cycle at G0/G1 stage and significantly decreased cyclin D1 expression in thyroid cells. Moreover, lncRNA PANCAR could induce cell apoptosis of thyroid cells.

PANDAR has been implicated in the development of various cancers, including colorectal cancer, where the high expression of PANDAR indicates a poor prognosis and promotes metastasis by EMT pathway (Lu et al., 2017[12]), and increased expression of PANDAR predicts a poor prognosis in gastric cancer (Ma et al., 2016[13]). Moreover, low expression of long noncoding RNA PANDAR predicts a poor prognosis of non-small cell lung cancer and affects cell apoptosis by regulating Bcl-2 (Han et al., 2015[7]). In this study, the data showed that PANDAR was significantly upregulated in thyroid cancer tissues compared to its adjacent normal tissues, as well as in the five kinds of tumor cell lines (Figure 1(Fig. 1)). Therefore, we speculated that PANDAR overexpression may be correlated with thyroid cancer pathogenesis.

Accordingly, the effects of PANDAR expression on thyroid cancer cell proliferation were further analyzed using siRNA-mediated gene silencing. Our results showed that thyroid cancer cell proliferation and colony formation were significantly suppressed by siRNA-PANDAR transfection (Figure 2(Fig. 2)), indicating that PANDAR could promote thyroid cancer cell proliferation. Consequently, we further analyzed the effects of PANDAR expression on thyroid cell cycle and cell cycle-associated protein expression. Our data showed that thyroid cell cycle was arrested at G0/G1 stage by silencing PANDAR, and cell cycle-associated protein Chk1, Cdc25A and cyclin D1 expression was significantly decreased (Figure 3(Fig. 3)). 

Defective apoptosis is one of the hallmarks of cancer cells and increasing cell apoptosis could prevent cancer progression. Knockdown of PANDAR by siRNA induced apoptosis of thyroid cells by decreasing Bcl-2 expression and activating Bax (Figure 4(Fig. 4)), indicating that PANDAR inhibition could induce thyroid cancer cell apoptosis.

In summary, the findings from our study demonstrated that the downregulation of the PANDAR expression inhibits cell proliferation, arrests cell cycle and facilitates apoptosis. Thus, PANDAR could be a promising therapeutic target and novel molecular biomarker for TC.

## Conflict of interest

The authors declare that they have no conflict of interest.

## Funding

This study was supported by Chongqing foundational and perspective research program (Grant No.: cstc2016jcyjA0579).
